# Supplementary material for: A High Resolution Mass Spectrometry Study Reveals the Potential of Disulfide Formation in Human Mitochondrial Voltage-Dependent Anion Selective Channel Isoforms (hVDACs)
Source: Int J Mol Sci. 2020 Feb 21;21(4):1468. doi: 10.3390/ijms21041468 (PMC7073118; doi:10.3390/ijms21041468)
Supplement: Supplementary file 1 [file ijms-21-01468-s001.zip › ijms-686340-supplementary.docx]

**Supplementary Materials:**

A High Resolution Mass Spectrometry Study Reveals the Potential of Disulfide Formation in Human Mitochondrial Voltage-Dependent Anion Selective Channel Isoforms (hVDACs)

Maria G.G. Pittalà ^1^, Rosaria Saletti ^2,^*, Simona Reina ^3^, Vincenzo Cunsolo ^2^ , Vito De Pinto ^1,^*, and Salvatore Foti ^2^

^1^ Department of Biomedical and Biotechnological Sciences, University of Catania, Via S. Sofia 64, 95123 Catania, Italy; [marinella.pitt@virgilio.it](mailto:marinella.pitt@virgilio.it)

^2^ Department of Chemical Sciences, Organic Mass Spectrometry Laboratory, University of Catania, Viale A. Doria 6, 95125 Catania, Italy; [vcunsolo@unict.it](mailto:vcunsolo@unict.it) (V.C.); [sfoti@unict.it](mailto:sfoti@unict.it) (S.F.)

^3^ Department of Biological, Geological and Environmental Sciences, Section of Molecular Biology, University of Catania, Viale A. Doria 6, 95125 Catania, Italy; simonareina@yahoo.it

***** Correspondence: [rsaletti@unict.it](mailto:rsaletti@unict.it) (R.S.); Tel: +39 095 738 5026; [vdpbiofa@unict.it](mailto:vdpbiofa@unict.it) (V.D.P.); Tel: +39 095 7384244

Received: 19 December 2019; Accepted: 18 February 2020; Published: date

**Supplementary Fig.1**


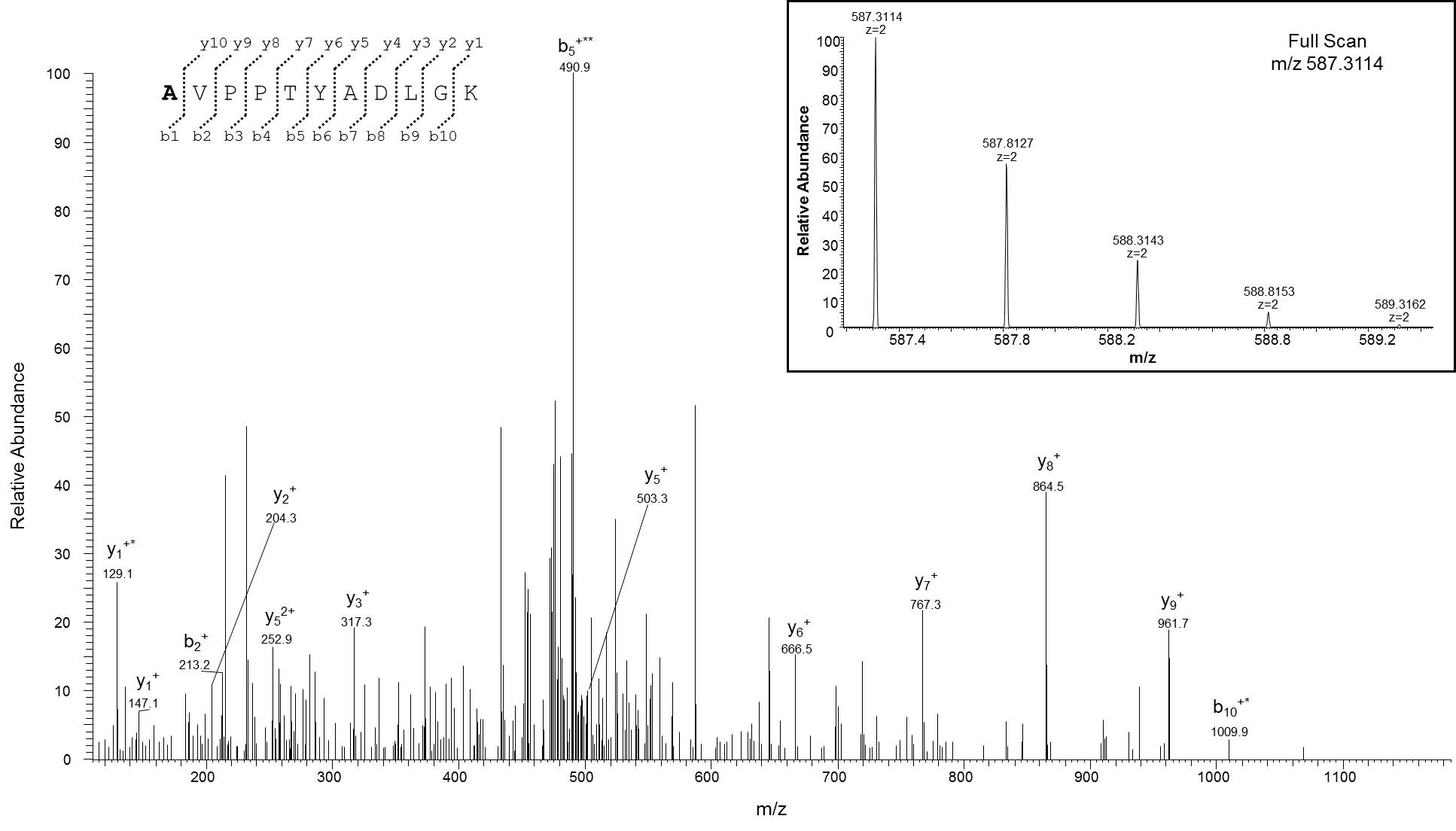


**Supplementary Figure S1.** MS/MS spectrum of the doubly charged molecular ion at m/z 587.3114 (calculated 587.3111) of the N-terminal acetylated tryptic peptide of VDAC1 from HAP1 cells*.* The inset shows the full scan mass spectrum of molecular ion. Fragment ions originated from the neutral loss of H_2_O are indicated by an asterisk.

**Supplementary Fig.2**


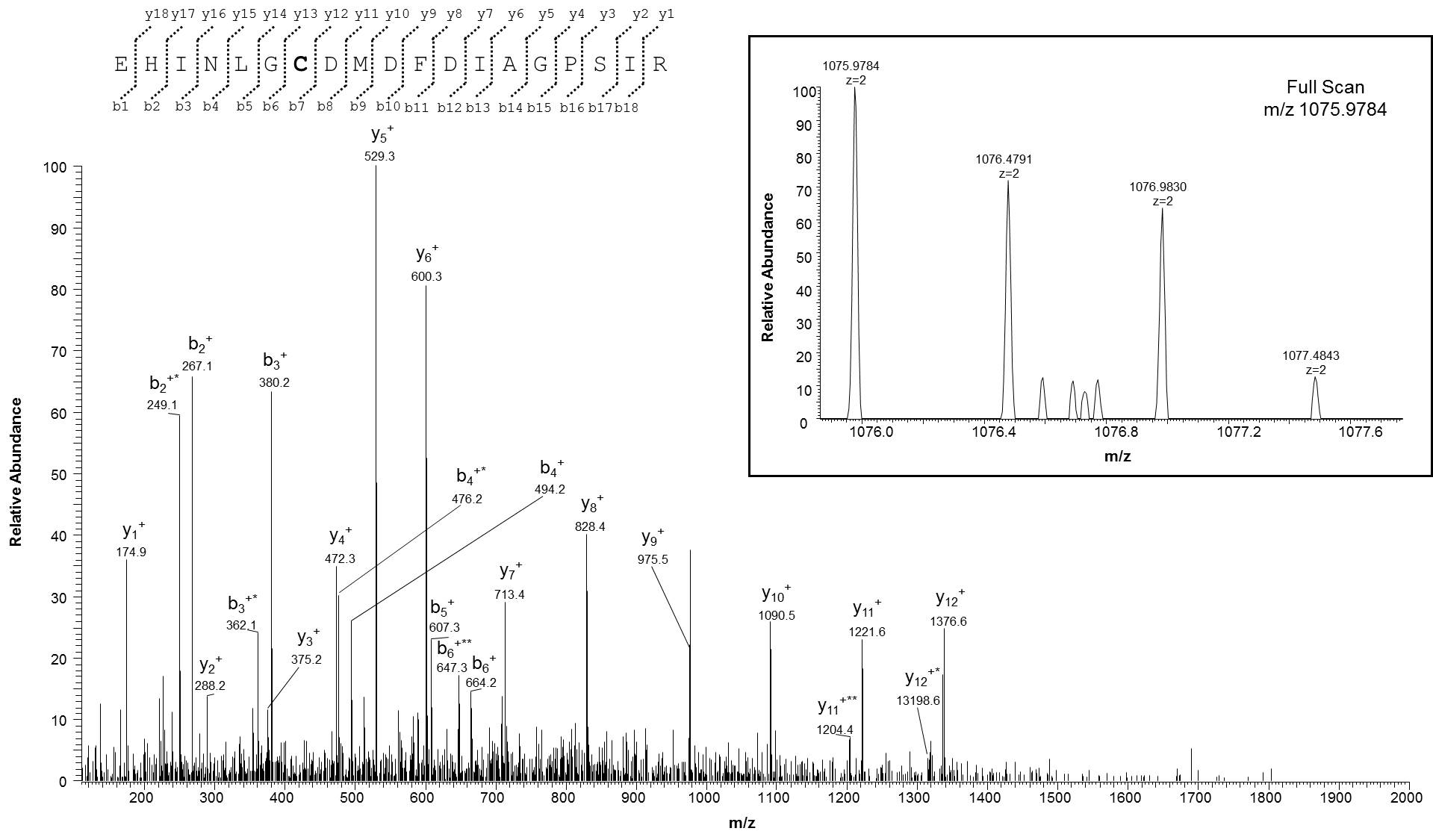


**Supplementary Figure S2.** MS/MS spectrum of the doubly charged molecular ion at m/z 1075.9784 (calculated 1075.9780) of the VDAC1 tryptic peptide from HAP1 cells containing containing cysteine residue 127 in the form of sulfonic acid. The inset shows the full scan mass spectrum of molecular ion. Fragment ions originated from the neutral loss of H_2_O are indicated by an asterisk. Fragment ions originated from the neutral loss of NH_3_ are indicated by two asterisks.

**Supplementary Fig.3**


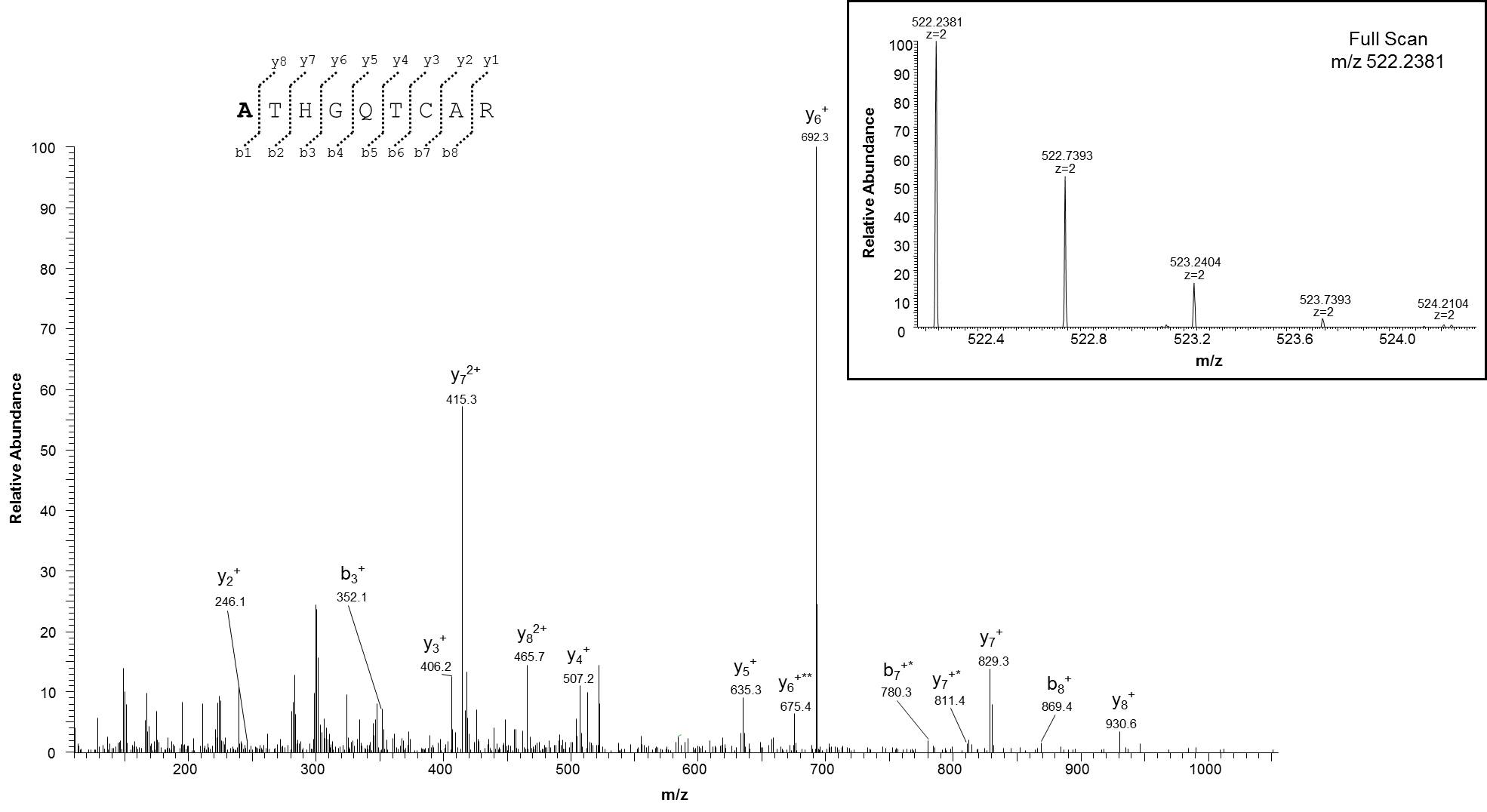


**Supplementary Figure S3**. MS/MS spectrum of the doubly charged molecular ion at m/z 522.2381 (calculated 522.2380) of the N-terminal acetylated tryptic peptide of VDAC2 from HAP1 cells*.* The inset shows the full scan mass spectrum of molecular ion. Fragment ions originated from the neutral loss of H_2_O are indicated by an asterisk. Fragment ion originated from the neutral loss of NH_3_ is indicated by two asterisks.

**Supplementary Fig.4**


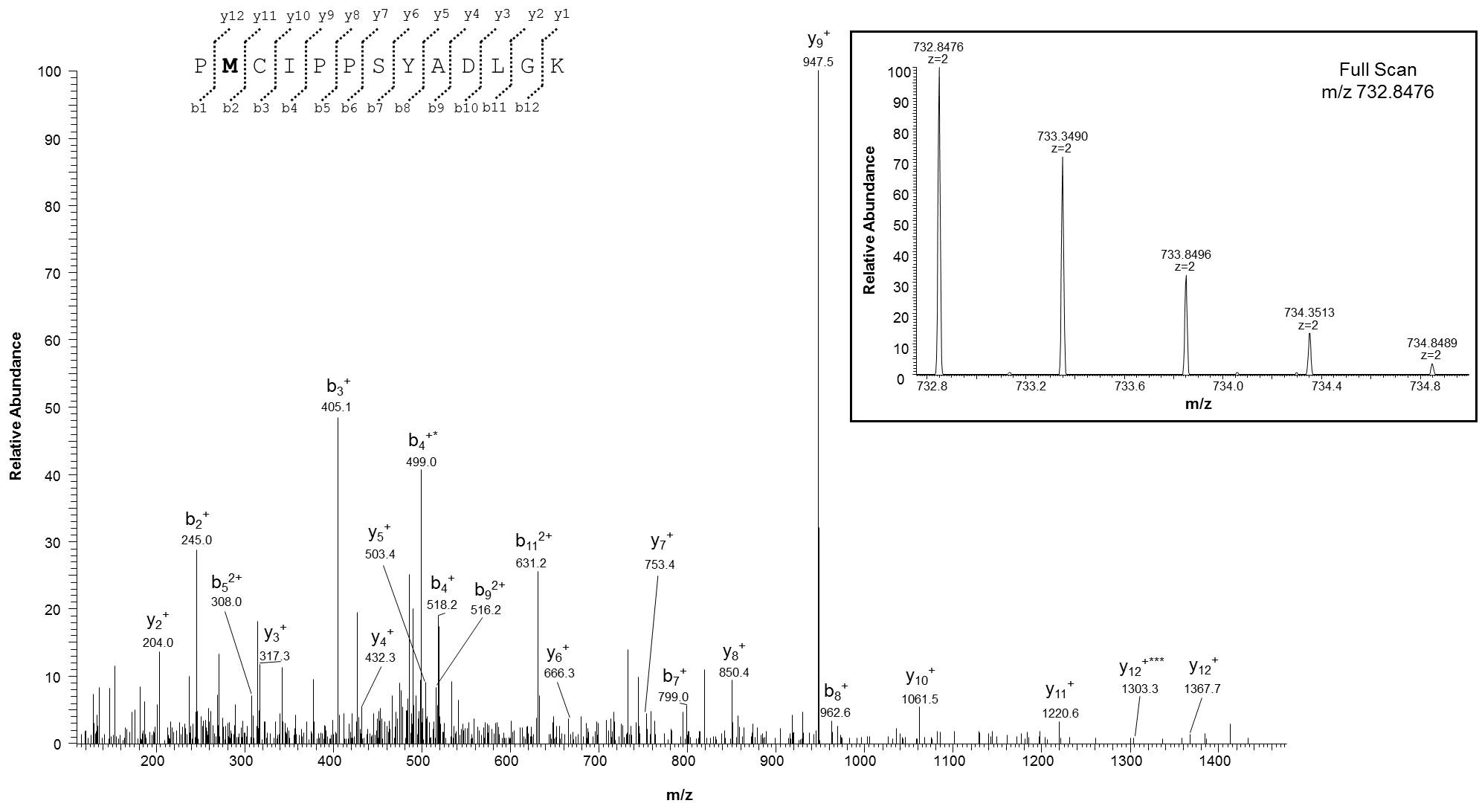


**Supplementary Figure S4.** MS/MS spectrum of the doubly charged molecular ion at m/z 732.8476 (calculated 732.8470) of the VDAC2 tryptic peptide from HAP1 cells containing methionine residue 12 in the oxidized form of methionine sulfoxide. The inset shows the full scan mass spectrum of molecular ion. Fragment ion originated from the neutral loss of H_2_O is indicated by an asterisk. Fragment ion originated from the neutral loss of methanesulfenic acid (CH_2_SOH, 64 Da) is indicated by three asterisks.

**Supplementary Fig.5**


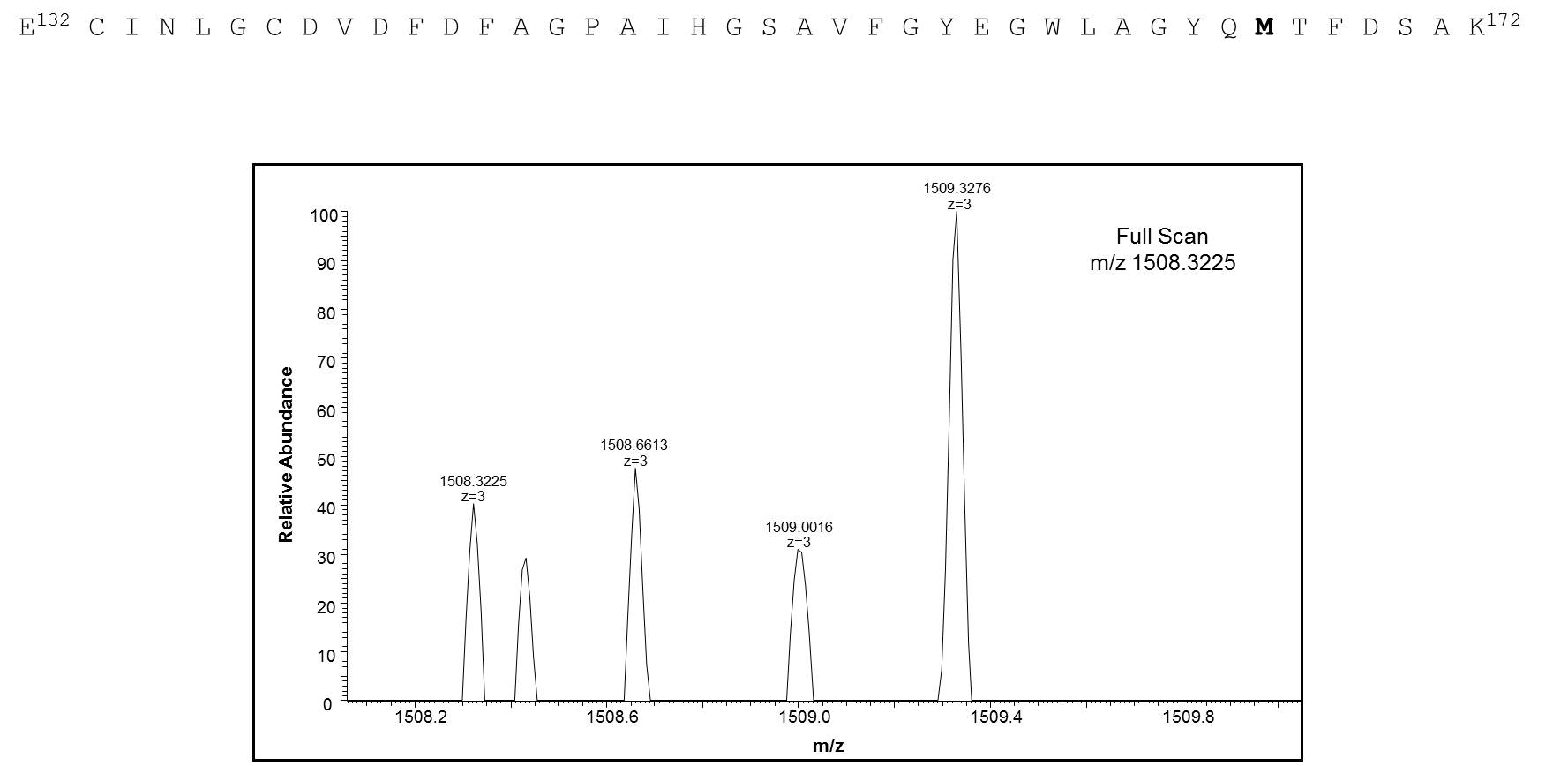


**Supplementary Figure S5.** Full scan mass spectrum of the triply charged molecular ion at m/z 1058.3225 (calculated 1058.3244) of the VDAC2 tryptic peptide from HAP1 cells containing methionine residue 166 in the oxidized form of methionine sulfoxide and one cysteine residue trioxidized.

**Supplementary Fig.6A**


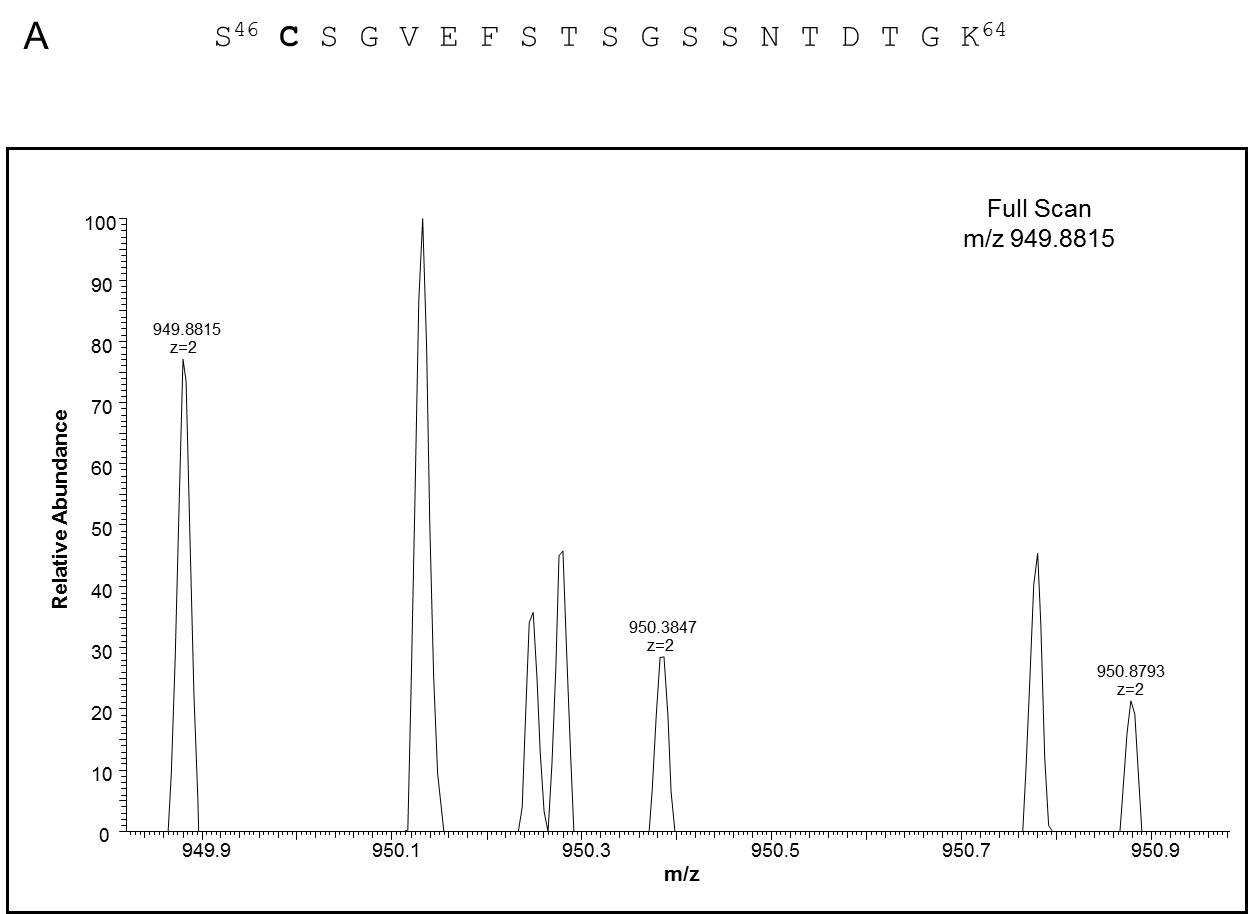


**Supplementary Figure S6A.** Full scan mass spectrum of the doubly charged molecular ion at m/z 949.8815 (calculated 949.8817) of the VDAC2 tryptic peptide from HAP1 cells containing cysteine residue 47 in the form of sulfonic acid.

**Supplementary Fig.6B**


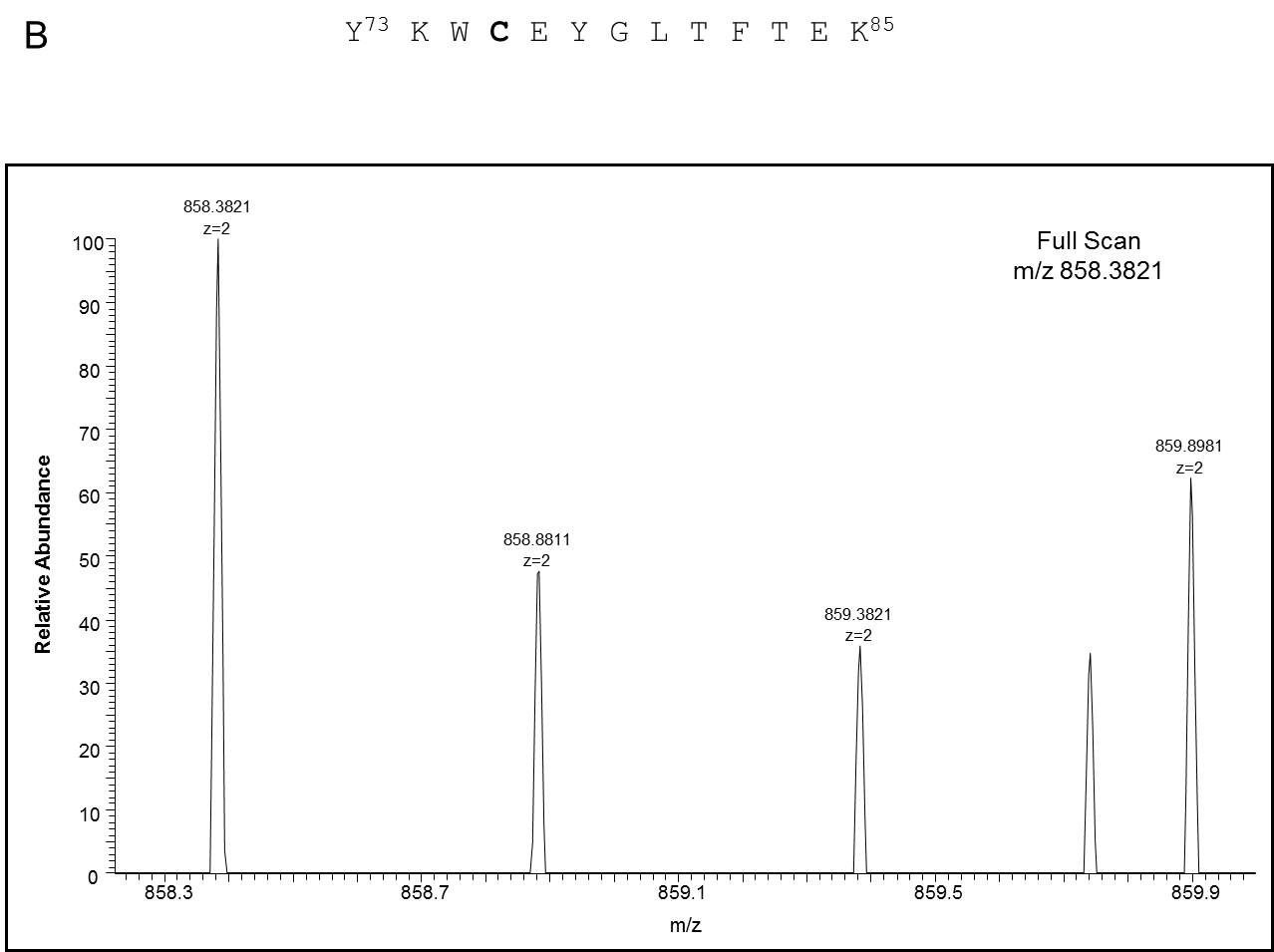


**Supplementary Figure S6B.** Full scan mass spectrum of the doubly charged molecular ion at m/z 858.3821 (calculated 858.3849) of the VDAC2 tryptic peptide from HAP1 cells containing cysteine residue 76 in the form of sulfonic acid.

**Supplementary Fig.6C**


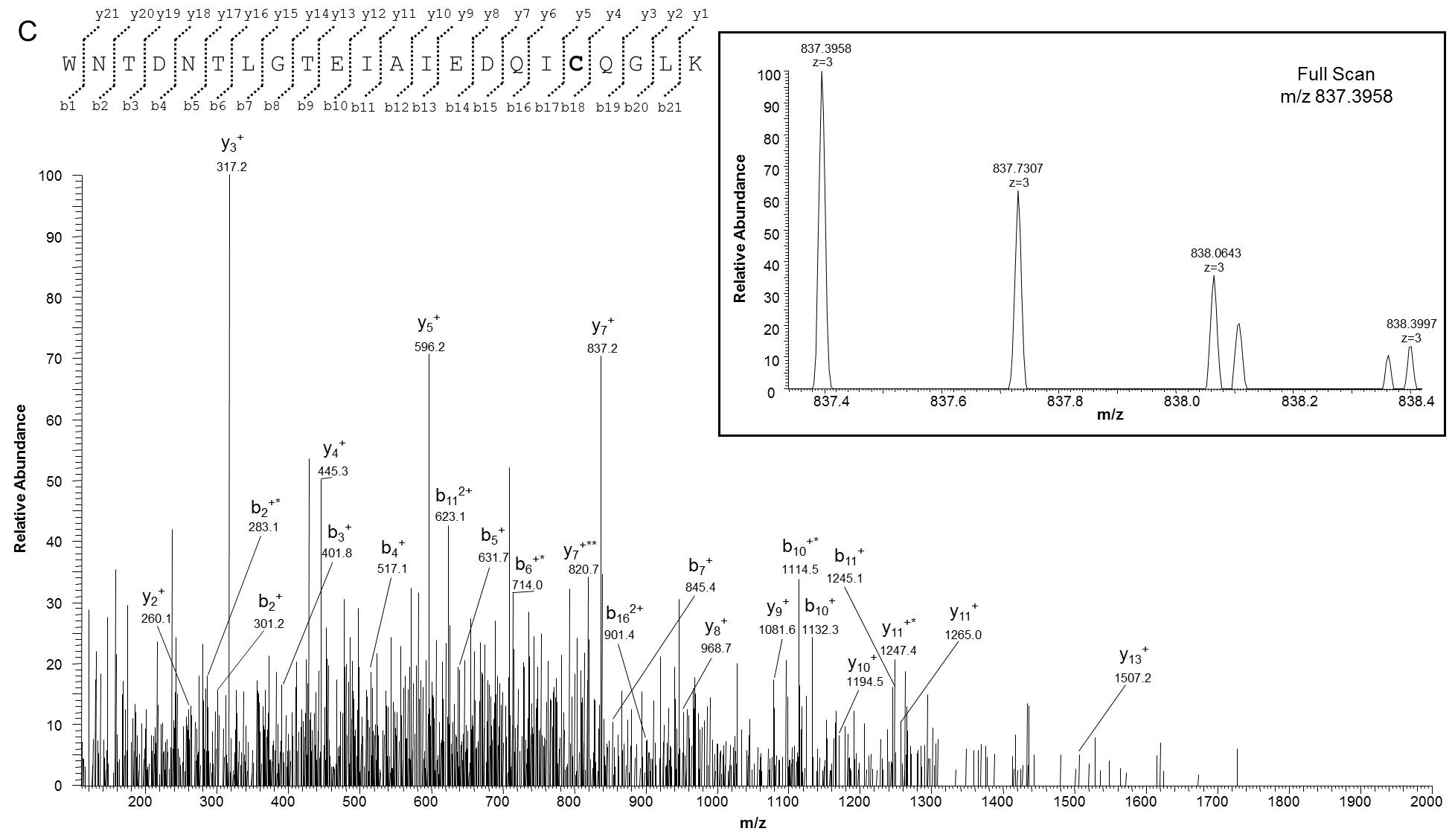


**Supplementary Figure S6C.** MS/MS spectrum of the triply charged molecular ion at m/z 837.3958 (calculated 837.3957) of the VDAC2 tryptic peptide from HAP1 cells containing cysteine residue 103 in the form of sulfonic acid. The inset shows the full scan mass spectrum of molecular ion. Fragment ions originated from the neutral loss of H_2_O are indicated by an asterisk. Fragment ion originated from the neutral loss of NH_3_ is indicated by two asterisks.

**Supplementary Fig.6D**


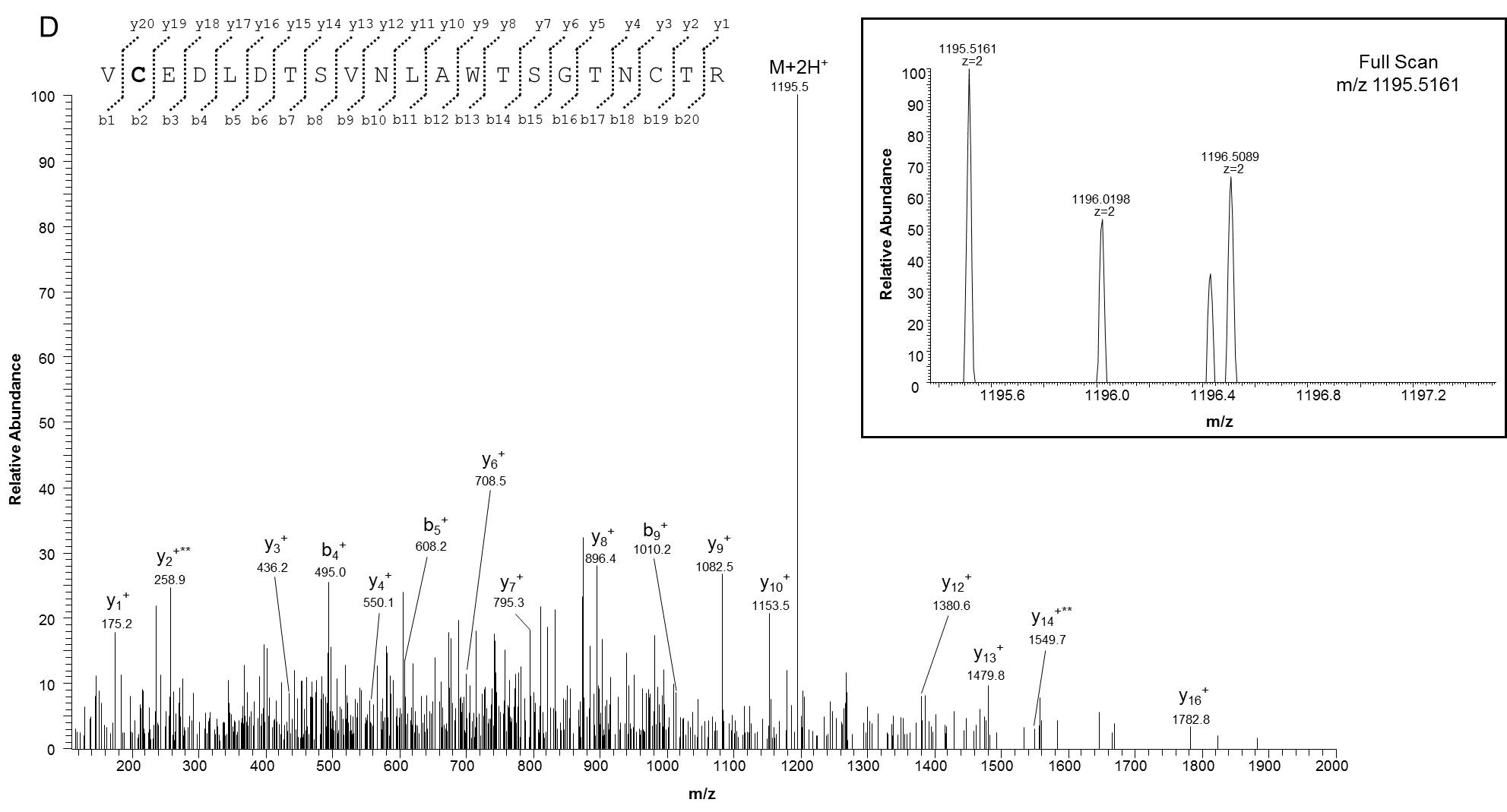


**Supplementary Figure S6D.** Full scan mass spectrum of the doubly charged molecular ion at m/z 1195.5161 (calculated 1195.5157) of the VDAC2 tryptic peptide from HAP1 cells containing cysteine residue 210 in the form of sulfonic acid. The inset shows the full scan mass spectrum of molecular ion. Fragments ions originated from the neutral loss of NH_3_ are indicated by two asterisks.

**Supplementary Fig.7**


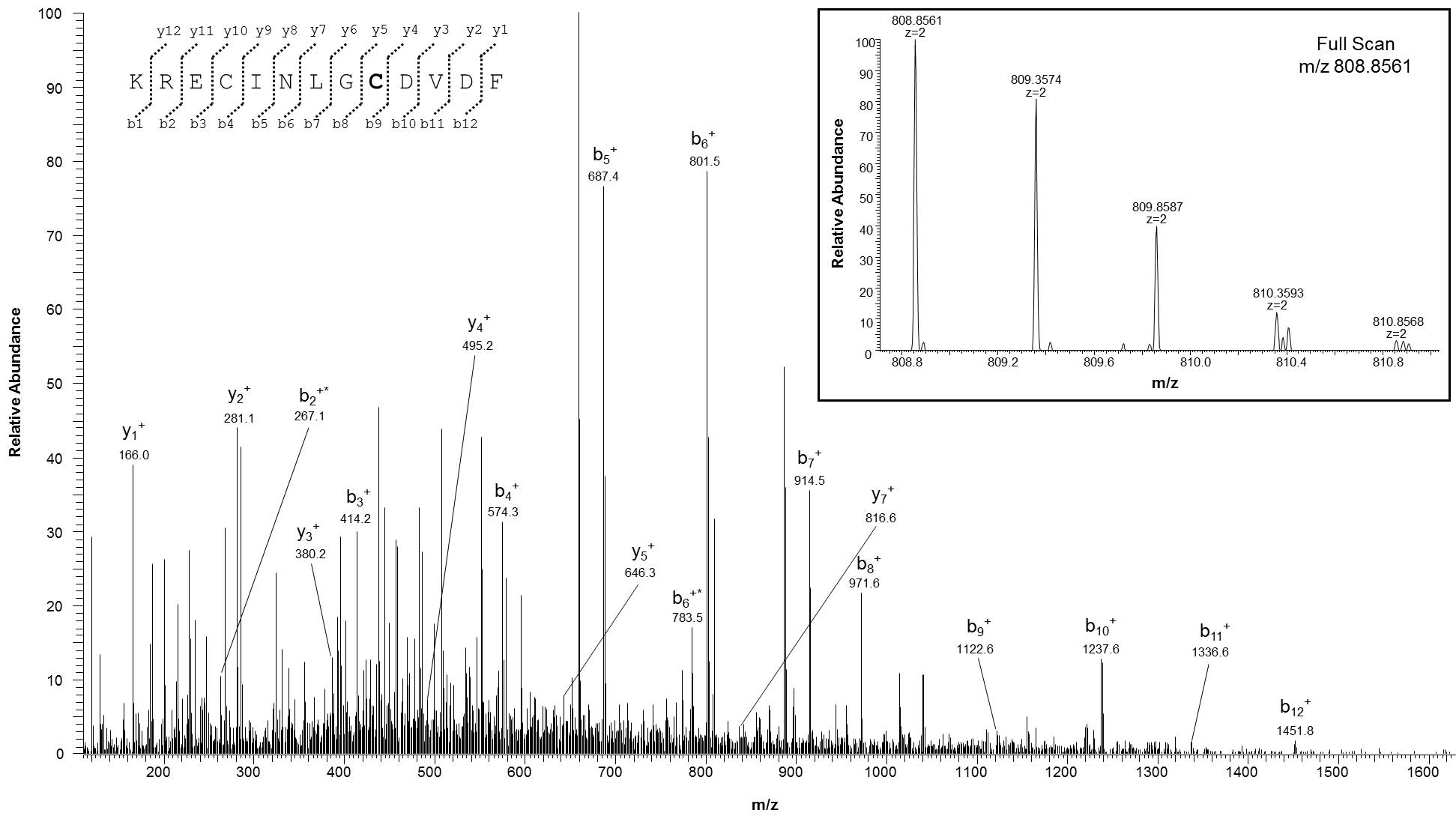


**Supplementary Figure S7.** MS/MS spectrum of the doubly charged molecular ion at m/z 808.8561 (calculated 808.8565) of the VDAC2 chymotryptic peptide from HAP1 cells containing cysteine residue 138 in the form of sulfonic acid. The inset shows the full scan mass spectrum of molecular ion. Fragment ions originated from the neutral loss of H_2_O are indicated by an asterisk.

**Supplementary Fig.8**


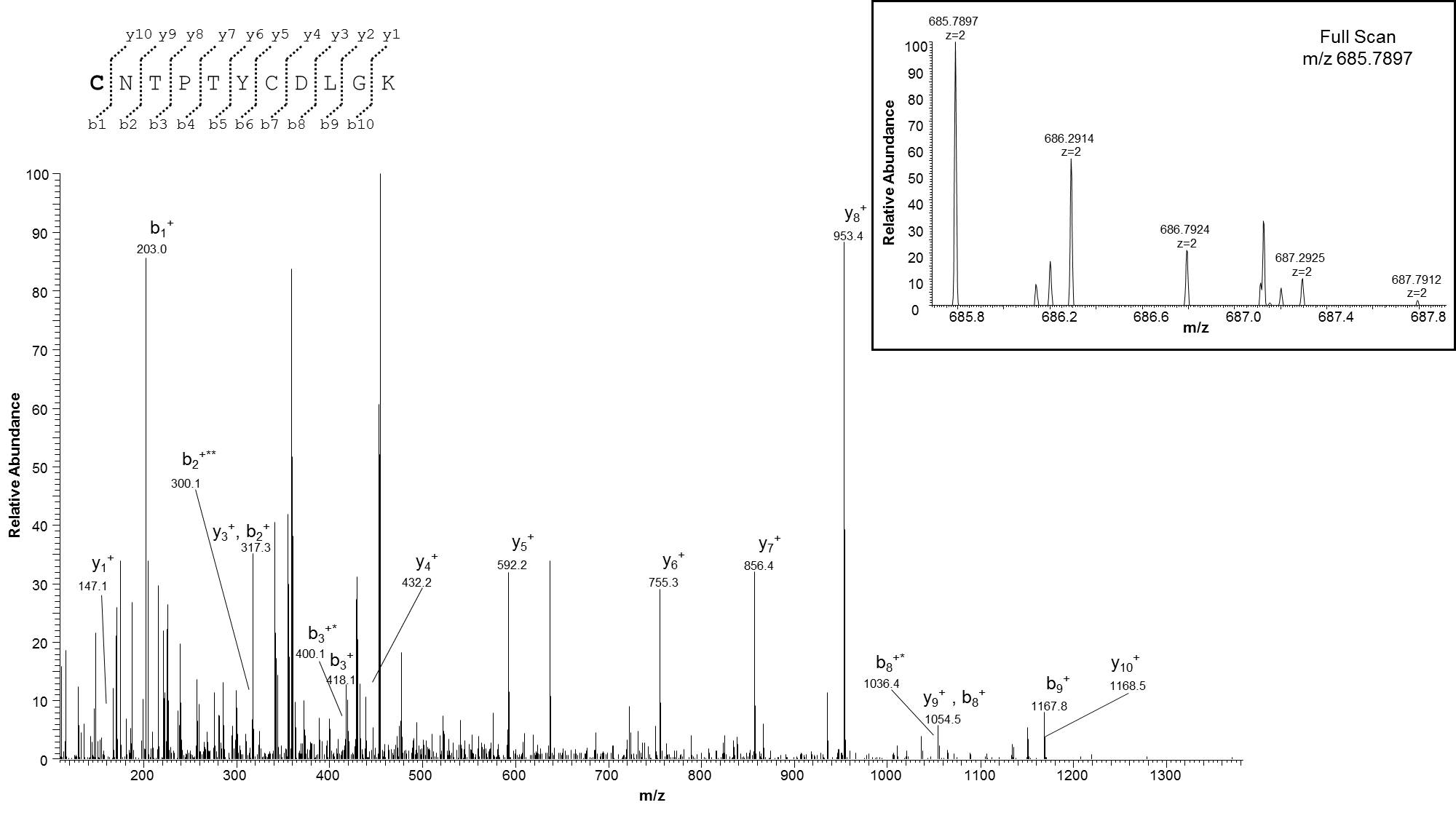


**Supplementary Figure S8.** MS/MS spectrum of the doubly charged molecular ion at m/z 685.7897 (calculated 685.7894) of the N-terminal acetylated tryptic peptide of VDAC3 from HAP1 cells with Cys^2^ and Cys^8^ in the carboxyamidomethylated form*.* The inset shows the full scan mass spectrum of molecular ion. Fragment ions originated from the neutral loss of H_2_O are indicated by an asterisk. Fragment ion originated from the neutral loss of NH_3_ is indicated by two asterisks.

**Supplementary Fig.9A**


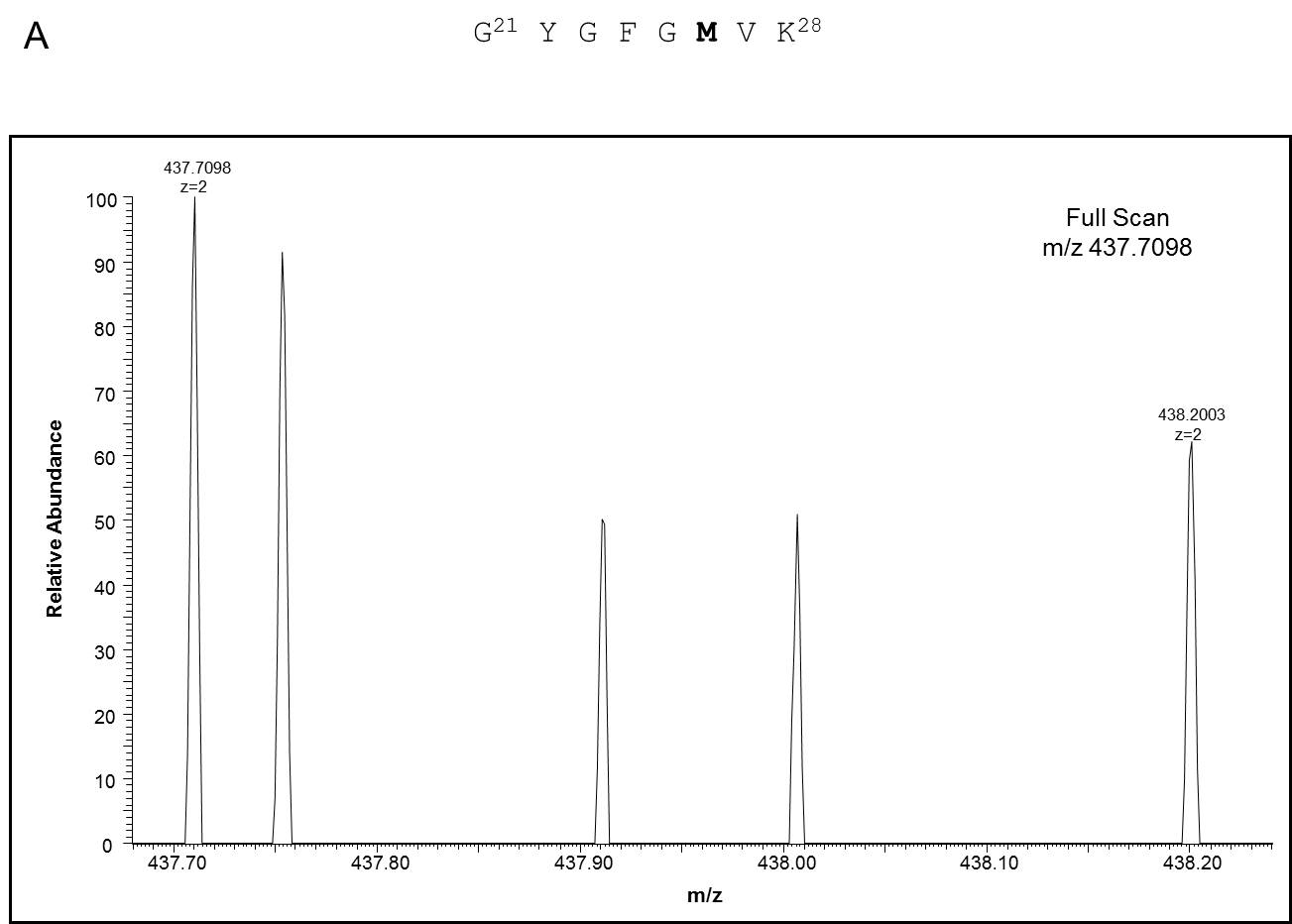


**Supplementary Figure S9A.** MS spectrum of the doubly charged molecular ion at m/z 437.7098 (calculated 437.7103) of the VDAC3 tryptic peptide from HAP1 cells containing methionine residue 26 in the oxidized form of methionine sulfoxide.

**Supplementary Fig.9B**


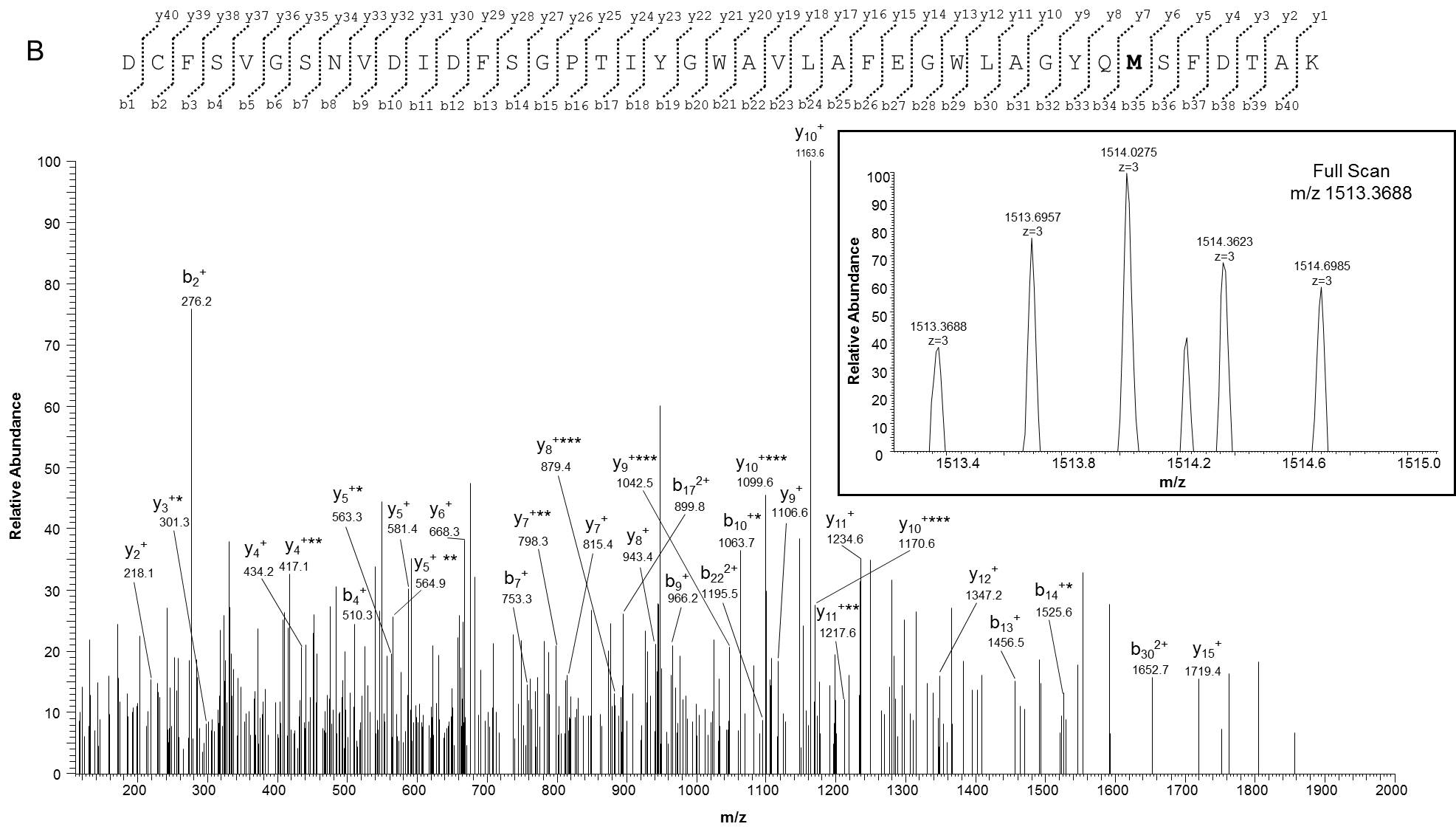


**Supplementary Figure S9B.** MS/MS spectrum of the triply charged molecular ion at m/z 1513.3688 (calculated 1513.3596) of the VDAC3 tryptic peptide from HAP1 cells containing methionine residue 155 in the oxidized form of methionine sulfoxide. The inset shows the full scan mass spectrum of molecular ion. Fragment ions originated from the neutral loss of H_2_O are indicated by an asterisk. Fragment ions originated from the neutral loss of NH_3_  are indicated by two asterisks. Fragment ions originated from the neutral loss of methanesulfenic acid (CH_2_SOH, 64 Da) are indicated by three asterisks.

**Supplementary Fig.9C**


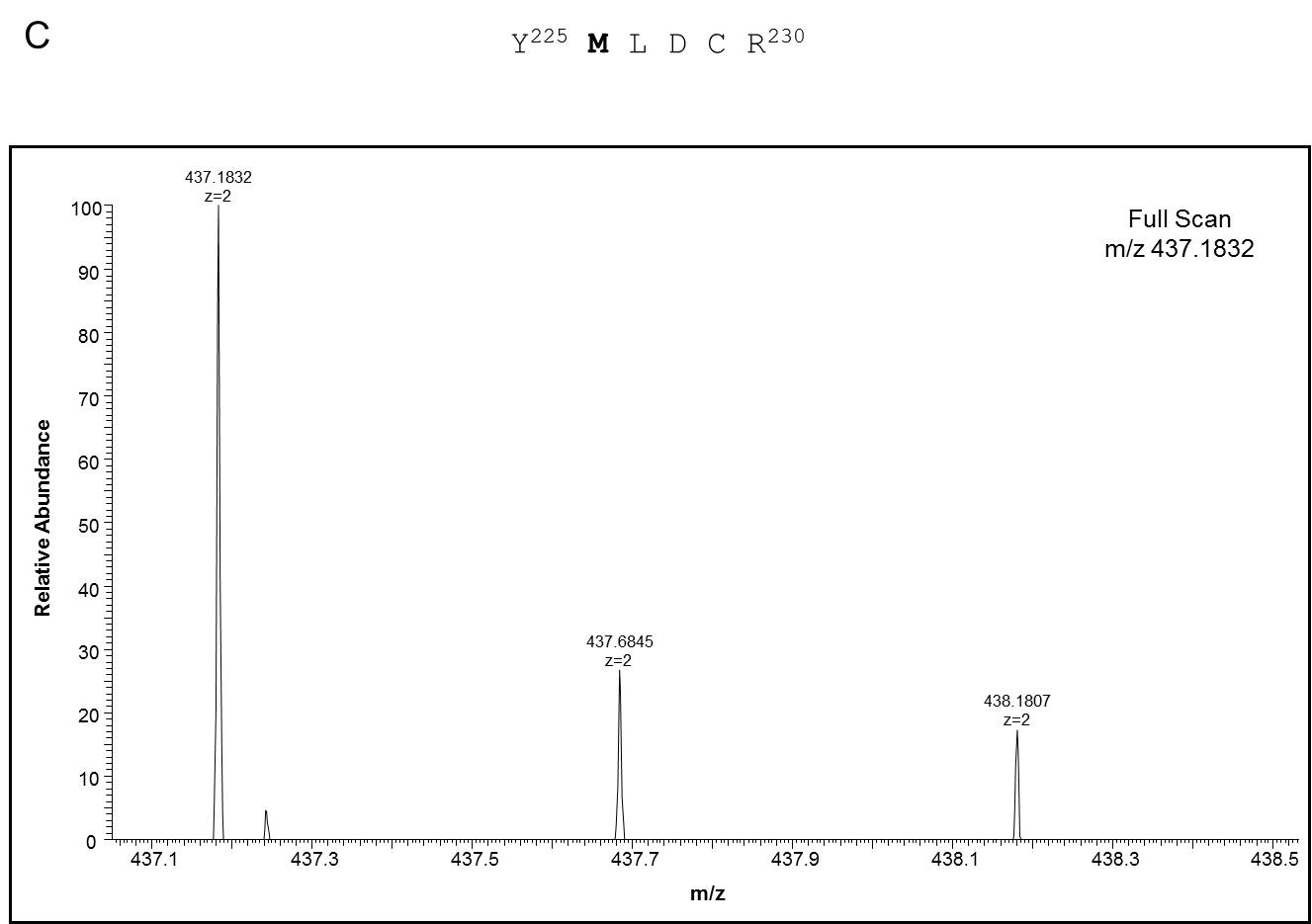


**Supplementary Figure S9C.** Full scan mass spectrum of the doubly charged molecular ion at m/z 437.1832 (calculated 437.1836) of the VDAC3 tryptic peptide from HAP1 cells containing methionine residue 226 in the oxidized form of methionine sulfoxide.

**Supplementary Fig.10A**


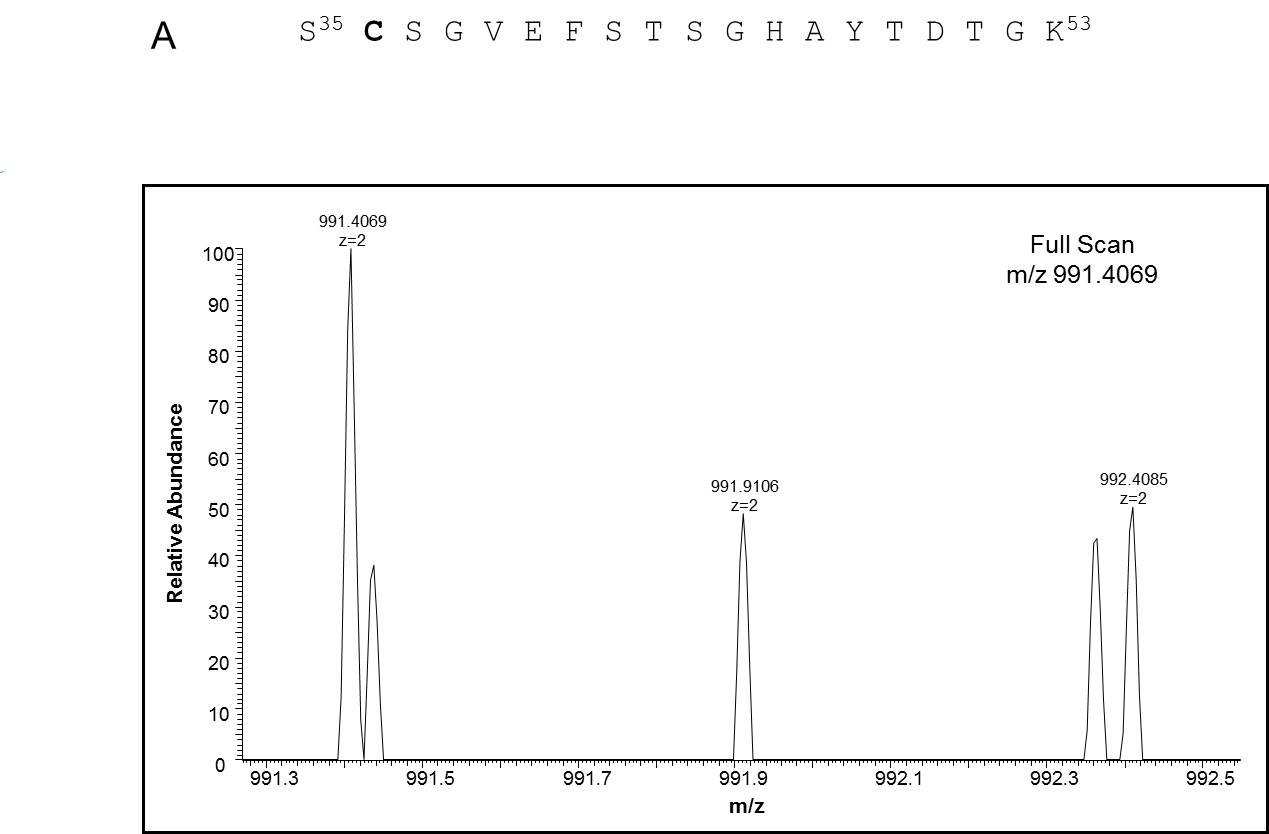


**Supplementary Figure S10A.** Full scan mass spectrum of the doubly charged molecular ion at m/z 991.4069 (calculated 991.4079) of the VDAC3 tryptic peptide from HAP1 cells containing cysteine residue 36 in the form of sulfonic acid.

**Supplementary Fig.10B**


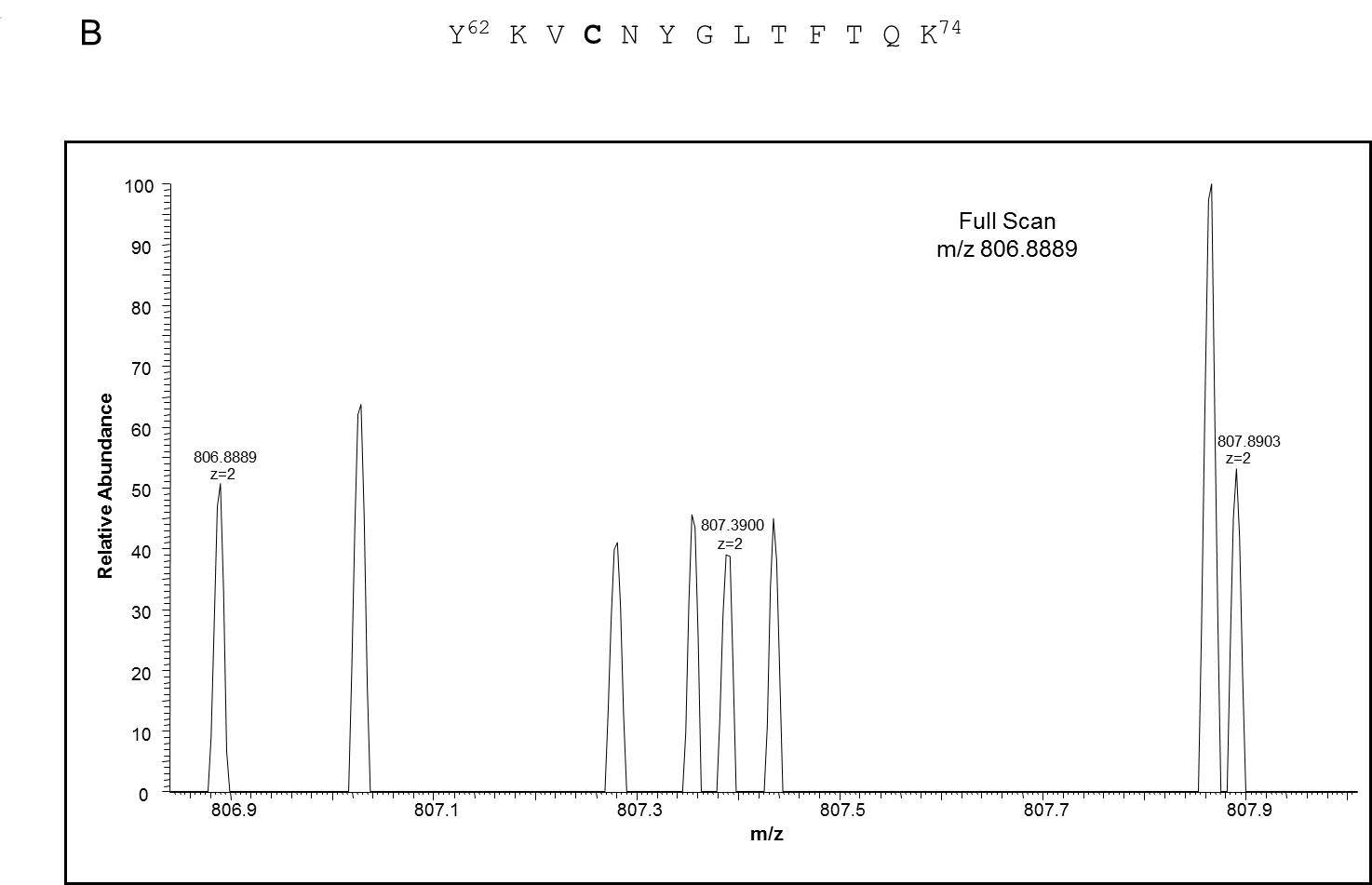


**Supplementary Figure S10B.** Full scan mass spectrum of the doubly charged molecular ion at m/z 806.8889 (calculated 806.8877) of the VDAC3 tryptic peptide from HAP1 cells containing cysteine residue 65 in the form of sulfonic acid.

**Supplementary Fig.11**


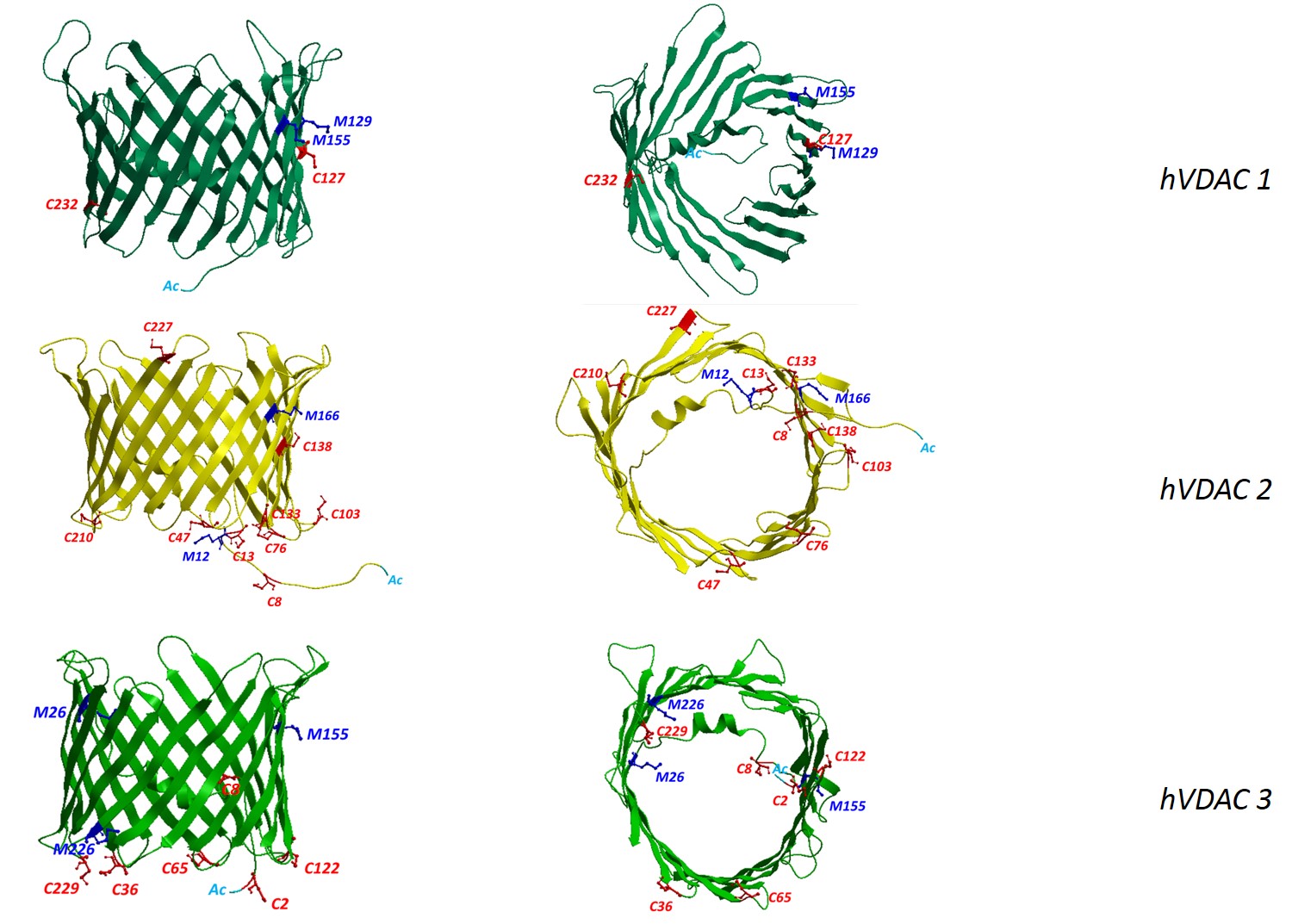
**Supplementary Figure S11.** Side and top views of human VDACs where the cysteine and methionine positions have been indicated. The structures are predicted by homology modelling, using hVDAC1 structure (pdb: 5XDO) as a template.

**Supplementary Tables**

**Table S1. Tryptic peptides found in hVDAC1 after DTT reduction and carboxyamidomethylation.**

Retention time, experimentally measured and calculated monoisotopic m/z of the molecular ions, position in the sequence and peptide sequence of fragments present in the tryptic digest of reduced and carboxyamidomethylated hVDAC1 are reported. All sequences were confirmed by MS/MS. These sequences were used to build the sequence coverage reported in Figure 1.

| **Frag.**  **n.** | **Rt**  **(min)** | **Monoisotopic m/z** | | **Position in the sequence** | **Peptide sequence** |
| --- | --- | --- | --- | --- | --- |
|  |  | **Measured** | **Calculated** |  |  |
| 1 | 52.77 | 587.3114 (+2) | 587.3111 | 2-12 | *AVPPTYADLGK |
| 2 | 77.18 | 744.3970 (+2) | 744.3963 | 2-15 | *AVPPTYADLGKSAR |
| 3 | 80.15 | 693.3687 (+3) | 693.3687 | 2-20 | *AVPPTYADLGKSARDVFTK |
| 4 | 79.64 | 462.2510 (+2) | 462.2509 | 13-20 | SARDVFTK |
| 5 | 82.18 | 586.9897 (+3) | 586.9894 | 13-28 | SARDVFTKGYGFGLIK |
| 6 | 82.96 | 743.4203 (+3) | 743.4194 | 13-32 | SARDVFTKGYGFGLIKLDLK |
| 7 | 74.53 | 638.6965 (+3) | 638.6960 | 16-32 | DVFTKGYGFGLIKLDLK |
| 8 | 71.47 | 427.7420 (+2) | 427.7422 | 21-28 | GYGFGLIK |
| 9 | 74.08 | 662.3874 (+2) | 662.3872 | 21-32 | GYGFGLIKLDLK |
| 10 | 69.86 | 730.3445 (+3) | 730.3450 | 33-53 | TKSENGLEFTSSGSANTETTK |
| 11 | 71.43 | 1002.1581 (+3) | 1002.1580 | 33-61 | TKSENGLEFTSSGSANTETTKVTGSLETK |
| 12 | 56.30 | 980.4427 (+2) | 980.4425 | 35-53 | SENGLEFTSSGSANTETTK |
| 13 | 70.52 | 925.7787 (+3) | 925.7771 | 35-61 | SENGLEFTSSGSANTETTKVTGSLETK |
| 14 | 53.90 | 417.7319 (+2) | 417.7320 | 54-61 | VTGSLETK |
| 15 | 71.71 | 565.2792 (+3) | 565.2789 | 62-74 | YRWTEYGLTFTEK |
| 16 | 71.28 | 687.8333 (+2) | 687.8324 | 64-74 | WTEYGLTFTEK |
| 17 | 69.62 | 1088.5302 (+2) | 1088.5295 | 75-93 | WNTDNTLGTEITVEDQLAR |
| 18 | 73.22 | 609.6602 (+3) | 609.6597 | 94-110 | GLKLTFDSSFSPNTGKK |
| 19 | 62.89 | 700.8389 (+2) | 700.8383 | 97-109 | LTFDSSFSPNTGK |
| 20 | 72.19 | 764.8867 (+2) | 764.8857 | 97-110 | LTFDSSFSPNTGKK |
| 21 | 81.07 | 433.2663 (+2) | 433.2663 | 114-120 | IKTGYKR |
| 22 | 74.14 | 1209.5944 (+2) | 1209.5936 | 140-161 | GALVLGYEGWLAGYQMNFETAK |
| 23 | 62.22 | 887.7768 (+3) | 887.7758 | 140-163 | GALVLGYEGWLAGYQMNFETAKSR |
| 24 | 79.64 | 728.8813 (+2) | 728.8808 | 162-174 | SRVTQSNFAVGYK |
| 25 | 58.47 | 607.3146 (+2) | 607.3142 | 164-174 | VTQSNFAVGYK |
| 26 | 72.70 | 867.4022 (+3) | 867.4015 | 175-197 | TDEFQLHTNVNDGTEFGGSIYQK |
| 27 | 81.33 | 763.0762 (+3) | 763.0766 | 198-218 | VNKKLETAVNLAWTAGNSNTR |
| 28 | 82.28 | 958.8586 (+3) | 958.8577 | 198-224 | VNKKLETAVNLAWTAGNSNTRFGIAAK |
| 29 | 72.32 | 649.3425 (+3) | 649.3412 | 201-218 | KLETAVNLAWTAGNSNTR |
| 30 | 81.60 | 845.1219 (+3) | 845.1222 | 201-224 | KLETAVNLAWTAGNSNTRFGIAAK |
| 31 | 65.55 | 606.6434 (+3) | 606.6429 | 202-218 | LETAVNLAWTAGNSNTR |
| 32 | 61.71 | 707.8189 (+2) | 707.8190 | 225-236 | YQIDPDA***C***FSAK |
| 33 | 75.07 | 1038.9291 (+3) | 1038.9306 | 237-266 | VNNSSLIGLGYTQTLKPGIKLTLSALLDGK |
| 34 | 71.82 | 603.3396 (+3) | 603.3390 | 257-274 | LTLSALLDGKNVNAGGHK |
| 35 | 66.29 | 947.5201 (+1) | 947.5197 | 275-283 | LGLGLEFQA |

^*^A: N-terminal acetylated; ***C***: cysteine carboxyamidomethylated.

**Table S2. Sulfur-modified peptides found in hVDAC1 tryptic digest after DTT reduction and carboxyamidomethylation.**

Retention time, experimentally measured and calculated monoisotopic m/z of the molecular ions, position in the sequence and peptide sequence of modified fragments present in the tryptic digest of reduced and carboxyamidomethylated hVDAC1 are reported. All sequences were confirmed by MS/MS. The sequence corresponding to the tryptic fragment 1 was used to build the sequence coverage reported in Figure 1.

| **Frag. n.** | **Rt (min)** | **Monoisotopic m/z** | | **Position in the sequence** | **Peptide sequence** |
| --- | --- | --- | --- | --- | --- |
|  |  | **Measured** | **Calculated** |  |  |
| 1 | 70.86 | 1075.9784 (+2) | 1075.9780 | 121-139 | EHINLG**C**DMDFDIAGPSIR |
| 2 | 66.31 | 1083.9756 (+2) | 1083.9754 | 121-139 | EHINLG**C**D**M**DFDIAGPSIR |
| 3 | 73.63 | 812.0634 (+3) | 812.0635 | 140-161 | GALVLGYEGWLAGYQ**M**NFETAK |

**M**: methionine sulfoxide; **C**: cysteine oxidized to sulfonic acid.

**Table S3. Chymotryptic peptides found in hVDAC1 after DTT reduction and carboxyamidomethylation.**

Retention time, experimentally measured and calculated monoisotopic m/z of the molecular ions, position in the sequence and peptide sequence of fragments present in the chymotryptic digest of reduced and carboxyamidomethylated hVDAC1 are reported. All sequences were confirmed by MS/MS. These sequences were used to build the sequence coverage reported in Figure 1.

| **Frag.**  **n.** | **Rt**  **(min)** | **Monoisotopic m/z** | | **Position in the sequence** | **Peptide sequence** |
| --- | --- | --- | --- | --- | --- |
|  |  | **Measured** | **Calculated** |  |  |
| 1 | 59.35 | 689.3504 (+1) | 689.3504 | 2-7 | *AVPPTY |
| 2 | 82.62 | 924.9778 (+2) | 924.9782 | 2-18 | *AVPPTYADLGKSARDVF |
| 3 | 83.19 | 1178.6160 (+1) | 1178.6164 | 8-18 | ADLGKSARDVF |
| 4 | 83.18 | 554.2932 (+2) | 554.2938 | 9-18 | DLGKSARDVF |
| 5 | 83.23 | 440.2374 (+2) | 440.2383 | 11-18 | GKSARDVF |
| 6 | 82.82 | 460.9050 (+3) | 460.9056 | 30-41 | DLKTKSENGLEF |
| 7 | 53.38 | 795.3524 (+1) | 795.3525 | 35-41 | SENGLEF |
| 8 | 66.64 | 820.9034 (+2) | 820.9029 | 41-58 | TSSGSANTETTKVTGSL |
| 9 | 53.38 | 666.9990 (+3) | 666.9996 | 41-61 | TSSGSANTETTKVTGSLETK |
| 10 | 82.47 | 1081.5264 (+2) | 1081.5266 | 41-62 | TSSGSANTETTKVTGSLETKY |
| 11 | 80.66 | 499.2637 (+2) | 499.2642 | 53-62 | VTGSLETKY |
| 12 | 82.61 | 754.3519 (+1) | 754.3524 | 63-67 | RWTEY |
| 13 | 59.87 | 881.9216 (+2) | 881.9213 | 76-91 | NTDNTLGTEITVEDQL |
| 14 | 78.55 | 995.4910 (+2) | 995.4904 | 76-93 | NTDNTLGTEITVEDQLAR |
| 15 | 82.57 | 1080.5427 (+2) | 1080.5431 | 76-95 | NTDNTLGTEITVEDQLARGL |
| 16 | 52.27 | 552.7734 (+2) | 552.7751 | 82-91 | GTEITVEDQL |
| 17 | 76.55 | 666.3443 (+2) | 666.3443 | 82-93 | GTEITVEDQLAR |
| 18 | 52.92 | 703.2937 (+1) | 703.2939 | 98-103 | TFDSSF |
| 19 | 83.58 | 436.9262 (+3) | 436.9265 | 107-118 | TGKKNAKIKTGY |
| 20 | 77.69 | 414.7321 (+2) | 414.7329 | 132-139 | DIAGPSIR |
| 21 | 82.57 | 535.3035 (+2) | 535.3042 | 132-142 | DIAGPSIRGAL |
| 22 | 82.68 | 666.3523 (+2) | 666.3519 | 130-142 | DFDIAGPSIRGAL |
| 23 | 71.00 | 823.3994 (+1) | 823.3990 | 143-149 | VLGYEGW |
| 24 | 83.04 | 814.9050 (+2) | 814.9055 | 156-169 | NFETAKSRVTQSNF |
| 25 | 82.72 | 684.3497 (+2) | 684.3499 | 158-169 | ETAKSRVTQSNF |
| 26 | 82.94 | 420.7684 (+2) | 420.7692 | 196-202 | **Q**KVNKKL |
| 27 | 83.05 | 489.9556 (+3) | 489.9564 | 196-208 | **Q**KVNKKLETAVNL |
| 28 | 84.04 | 508.3116 (+2) | 508.3115 | 200-208 | KKLETAVNL |
| 29 | 79.79 | 484.2333 (+2) | 484.2338 | 211-219 | TAGNSNTRF |
| 30 | 52.51 | 965.4039 (+1) | 965.4038 | 226-233 | QIDPDA***C***F |
| 31 | 69.89 | 948.3775 (+1) | 948.3773 | 226-233 | **Q**IDPDA***C***F |
| 32 | 76.02 | 460.2459 (+2) | 460.2464 | 234-242 | SAKVNNSSL |
| 33 | 82.82 | 493.3060 (+2) | 493.3062 | 248-256 | TQTLKPGIK |
| 34 | 83.18 | 549.8477 (+2) | 549.8482 | 248-257 | TQTLKPGIKL |
| 35 | 83.32 | 435.2939 (+2) | 435.2951 | 250-257 | TLKPGIKL |
| 36 | 83.18 | 605.3194 (+2) | 605.3209 | 264-275 | DGKNVNAGGHKL |
| 37 | 77.04 | 635.3403 (+1) | 635.3404 | 276-281 | GLGLEF |

^*^A: N-terminal acetylated; ***C***: cysteine carboxyamidomethylated.

**Table S4. Sulfur-modified peptides found in hVDAC1chymotryptic digest after DTT reduction and carboxyamidomethylation.**

Retention time, experimentally measured and calculated monoisotopic m/z of themolecular ions, position in the sequence and peptide sequence of modified fragments present in the tryptic digest of reduced and carboxyamidomethylated hVDAC1 are reported. All sequences were confirmed by MS/MS.

| **Frag. n.** | **Rt (min)** | **Monoisotopic m/z** | | **Position in the sequence** | **Peptide sequence** |
| --- | --- | --- | --- | --- | --- |
|  |  | **Measured** | **Calculated** |  |  |
| 1 | 83.08 | 547.9050 (+3) | 547.9052 | 119-131 | KREHINLG**C**D**M**DF |
| 2 | 83.37 | 817.7180 (+3) | 817.7184 | 119-139 | KREHINLG**C**D**M**DFDIAGPSIR |
| 3 | 64.06 | 546.2050 (+2) | 546.2051 | 123-131 | INLG**C**D**M**DF |

**M**: methionine sulfoxide; **C**: cysteine oxidized to sulfonic acid.

**Table S5. Tryptic peptides found in hVDAC2 after DTT reduction and carboxyamidomethylation.**

Retention time, experimentally measured and calculated monoisotopic m/z of the molecular ions, position in the sequence and peptide sequence of fragments identified in the tryptic digest of reduced and carboxyamidomethylated hVDAC2 are reported. All sequences were confirmed by MS/MS. These sequences were used to build the sequence coverage reported in Figure 4.

| **Frag.**  **n.** | **Rt**  **(min)** | **Monoisotopic m/z** | | **Position in the sequence** | **Peptide sequence** |
| --- | --- | --- | --- | --- | --- |
|  |  | **Measured** | **Calculated** |  |  |
| 1 | 20.60 | 522.2381 (+2) | 522.2380 | 2-10 | *ATHGQT***C***AR |
| 2 | 62.96 | 724.8497 (+2) | 724.8493 | 11-23 | PM***C***IPPSYADLGK |
| 3 | 69.39 | 580.6580 (+3) | 580.6579 | 24-39 | AARDIFNKGFGFGLVK |
| 4 | 52.32 | 636.3359 (+1) | 636.3352 | 27-31 | DIFNK |
| 5 | 73.66 | 721.3983 (+2) | 721.3955 | 27-39 | DIFNKGFGFGLVK |
| 6 | 73.41 | 633.0246 (+3) | 633.0243 | 27-43 | DIFNKGFGFGLVKLDVK |
| 7 | 72.70 | 824.4675 (+1) | 824.4665 | 32-39 | GFGFGLVK |
| 8 | 69.05 | 640.3745(+2) | 640.3741 | 32-43 | GFGFGLVKLDVK |
| 9 | 76.34 | 989.4697 (+3) | 989.4680 | 44-72 | TKS***C***SGVEFSTSGSSNTDTGKVTGTLETK |
| 10 | 50.57 | 954.4005 (+2) | 954.3998 | 46-64 | S***C***SGVEFSTSGSSNTDTGK |
| 11 | 62.32 | 913.0883 (+3) | 913.0871 | 46-72 | S***C***SGVEFSTSGSSNTDTGKVTGTLETK |
| 12 | 54.83 | 424.7398 (+2) | 424.7398 | 65-72 | VTGTLETK |
| 13 | 70.28 | 862.9037 (+2) | 862.9031 | 73-85 | YKW***C***EYGLTFTEK |
| 14 | 70.20 | 717.3246 (+2) | 717.3239 | 75-85 | W***C***EYGLTFTEK |
| 15 | 70.08 | 1260.1086 (+2) | 1260.1078 | 86-107 | WNTDNTLGTEIAIEDQI***C***QGLK |
| 16 | 63.73 | 714.8543 (+2) | 714.8539 | 108-120 | LTFDTTFSPNTGK |
| 17 | 72.29 | 519.6038 (+3) | 519.6034 | 108-121 | LTFDTTFSPNTGKK |
| 18 | 81.00 | 441.2637 (+2) | 441.2638 | 125-131 | IKSSYKR |
| 19 | 60.36 | 470.7354 (+2) | 470.7354 | 178-185 | NNFAVGYR |
| 20 | 73.51 | 843.3954 (+3) | 843.3945 | 186-208 | TGDFQLHTNVNDGTEFGGSIYQK |
| 21 | 64.20 | 800.3594 (+3) | 800.3583 | 209-229 | V***C***EDLDTSVNLAWTSGTN***C***TR |
| 22 | 67.28 | 627.6754 (+3) | 627.6754 | 230-247 | FGIAAKYQLDPTASISAK |
| 23 | 60.48 | 647.3386 (+2) | 647.3379 | 236-247 | YQLDPTASISAK |
| 24 | 72.52 | 1052.0819 (+2) | 1052.0815 | 248-267 | VNNSSLIGVGYTQTLRPGVK |
| 25 | 63.77 | 861.4634 (+2) | 861.4627 | 248-263 | VNNSSLIGVGYTQTLR |
| 26 | 70.64 | 508.8031 (+2) | 508.8030 | 268-277 | LTLSALVDGK |
| 27 | 73.06 | 560.3090 (+3) | 560.3089 | 278-294 | SINAGGHKVGLALELEA |
| 28 | 86.17 | 914.5200 (+1) | 914.5193 | 286-294 | VGLALELEA |

***C***: cysteine carboxyamidomethylated.

**Table S6. Sulfur-modified peptides found in hVDAC2 tryptic digest after DTT reduction and carboxyamidomethylation.**

Retention time, experimentally measured and calculated monoisotopic m/z of the molecular ions, position in the sequence and peptide sequence of modified fragments present in the tryptic digest of reduced and carboxyamidomethylated hVDAC2 are reported. The sequence corresponding to the tryptic fragment 6 was used to build the sequence coverage reported in Figure 4.

| **Frag. n.** | **Rt (min)** | **Monoisotopic m/z** | | **Position in the sequence** | **Peptide sequence** |
| --- | --- | --- | --- | --- | --- |
|  |  | **Measured** | **Calculated** |  |  |
| 1 | 70.99 | 830.3859 (+3) | 830.3842 | 2-23 | *ATHGQT***C***ARP**M*C***IPPSYADLGK |
| 2 | 58.95 | 732.8476 (+2) | 732.8470 | 11-23 | P**M*C***IPPSYADLGK |
| 3 | 34.88 | 949.8815 (+2) | 949.8817 | 46-64 | S**C**SGVEFSTSGSSNTDTGK |
| 4 | 50.32 | 858.3821 (+2) | 858.3849 | 73-85 | YKW**C**EYGLTFTEK |
| 5 | 64.20 | 837.3958 (+3) | 837.3957 | 86-107 | WNTDNTLGTEIAIEDQI**C**QGLK |
| 6 | 75.00 | 1502.9921 (+3) | 1502.9928 | 132-172 | ECINLGCDVDFDFAGPAIHGSAVFGYEGWLAGYQMTFDSAK^a^ |
| 7 | 74.86 | 1508.3225 (+3) | 1508.3244 | 132-172 | ECINLGCDVDFDFAGPAIHGSAVFGYEGWLAGYQ**M**TFDSAK^a^ |
| 8 | 58.38 | 1195.5161 (+2) | 1195.5157 | 209-229 | V**C**EDLDTSVNLAWTSGTNCTR |

^*^A: N-terminal acetylated; ***C***: cysteine carboxyamidomethylated;**M**: methionine sulfoxide; **C**: cysteine oxidized to sulfonic acid. ^a^One of the two cysteines of these peptides is trioxidized and one carboxyamidomethylated, but it was not possible to determine which one because the MS/MS spectrum was not obtained.

**Table S7. Chymotryptic peptides found in hVDAC2 after DTT reduction and carboxyamidomethylation.**

Retention time, experimentally measured and calculated monoisotopic m/z of the molecular ions, position in the sequence and peptide sequence of fragments present in the chymotryptic digest of reduced and carboxyamidomethylated hVDAC2 are reported. All sequences were confirmed by MS/MS. These sequences were used to build the sequence coverage reported in Figure 4.

| **Frag.**  **n.** | **Rt**  **(min)** | **Monoisotopic m/z** | | **Position in the sequence** | **Peptide sequence** |
| --- | --- | --- | --- | --- | --- |
|  |  | **Measured** | **Calculated** |  |  |
| 1 | 39.04 | 736.3335 (+1) | 736.3340 | 13-18 | ***C***IPPSY |
| 2 | 83.27 | 947.4795 (+2) | 947.4806 | 13-29 | ***C***IPPSYADLGKAARDIF |
| 3 | 83.33 | 588.8218 (+2) | 588.8228 | 19-29 | ADLGKAARDIF |
| 4 | 83.32 | 439.2476 (+2) | 439.2487 | 22-29 | GKAARDIF |
| 5 | 82.56 | 678.8268 (+2) | 678.8274 | 41-52 | DVKTKS***C***SGVEF |
| 6 | 66.10 | 806.8889 (+2) | 806.8872 | 53-69 | STSGSSNTDTGKVTGTL |
| 7 | 82.43 | 657.6551 (+3) | 657.6558 | 53-72 | STSGSSNTDTGKVTGTLETK |
| 8 | 82.44 | 1067.5112 (+2) | 1067.5115 | 53-73 | STSGSSNTDTGKVTGTLETKY |
| 9 | 82.56 | 785.3288 (+1) | 785.3292 | 74-78 | KW***C***EY |
| 10 | 84.62 | 491.2559 (+2) | 491.2556 | 79-86 | GLTFTEKW |
| 11 | 60.42 | 1017.9684 (+2) | 1017.9684 | 87-104 | NTDNTLGTEIAIEDQI***C***Q |
| 12 | 69.36 | 1103.0211 (+2) | 1103.0212 | 87-106 | NTDNTLGTEIAIEDQI***C***QGL |
| 13 | 62.59 | 773.8753 (+2) | 773.8751 | 93-106 | GTEIAIEDQI***C***QGL |
| 14 | 57.02 | 731.3260 (+1) | 731.3252 | 109-114 | TFDTTF |
| 15 | 83.55 | 527.9586 (+3) | 527.9586 | 115-129 | SPNTGKKSGKIKSSY |
| 16 | 83.58 | 428.5822 (+3) | 428.5827 | 118-129 | TGKKSGKIKSSY |
| 17 | 82.90 | 435.2398 (+2) | 435.2405 | 122-129 | SGKIKSSY |
| 18 | 80.60 | 414.2059 (+2) | 414.2065 | 143-150 | DFAGPAIH |
| 19 | 81.97 | 571.2858 (+2) | 571.2860 | 143-154 | DFAGPAIHGSAV |
| 20 | 82.62 | 644.8198 (+2) | 644.8202 | 143-155 | DFAGPAIHGSAVF |
| 21 | 82.90 | 487.2461 (+2) | 487.2467 | 185-192 | RTGDFQLH |
| 22 | 83.04 | 650.9693 (+3) | 650.9700 | 185-201 | RTGDFQLHTNVNDGTEF |
| 23 | 41.50 | 874.3983 (+1) | 874.3981 | 207-213 | **Q**KV***C***EDL |
| 24 | 42.96 | 695.8116 (+2) | 695.8120 | 207-218 | **Q**KV***C***EDLDTSVN |
| 25 | 66.97 | 704.3253 (+2) | 704.3252 | 207-218 | QKV***C***EDLDTSVN |
| 26 | 56.51 | 752.3541 (+2) | 752.3539 | 207-219 | **Q**KV***C***EDLDTSVNL |
| 27 | 82.63 | 889.4253 (+2) | 889.4255 | 207-221 | QKV***C***EDLDTSVNLAW |
| 28 | 42.36 | 648.3202 (+1) | 648.3204 | 214-219 | DTSVNL |
| 29 | 80.21 | 522.2326 (+2) | 522.2329 | 222-230 | TSGTN***C***TRF |
| 30 | 68.37 | 827.4156 (+1) | 827.4151 | 237-244 | **Q**LDPTASI |
| 31 | 48.45 | 844.4415 (+1) | 844.4416 | 237-244 | QLDPTASI |
| 32 | 43.57 | 931.4734 (+1) | 931.4736 | 237-245 | QLDPTASIS |
| 33 | 49.63 | 557.2931 (+2) | 557.2935 | 237-247 | **Q**LDPTASISAK |
| 34 | 69.78 | 565.8065 (+2) | 565.8068 | 237-247 | QLDPTASISAK |
| 35 | 70.92 | 672.3620 (+2) | 672.3624 | 237-249 | QLDPTASISAKVN |
| 36 | 78.57 | 872.9585 (+2) | 872.9580 | 237-253 | QLDPTASISAKVNNSSL |
| 37 | 66.80 | 1109.0743 (+2) | 1109.0740 | 237-258 | **Q**LDPTASISAKVNNSSLIGVGY |
| 38 | 83.37 | 1117.5881 (+2) | 1117.5873 | 237-258 | QLDPTASISAKVNNSSLIGVGY |
| 39 | 84.81 | 556.8430 (+2) | 556.8429 | 259-268 | TQTLRPGVKL |
| 40 | 91.33 | 903.5153 (+1) | 903.5151 | 269-277 | TLSALVDGK |
| 41 | 89.01 | 609.3410 (+2) | 609.3409 | 269-280 | TLSALVDGKSIN |
| 42 | 82.52 | 663.3441 (+2) | 663.3446 | 271-284 | SALVDGKSINAGGH |
| 43 | 83.37 | 861.9788 (+2) | 861.9790 | 271-288 | SALVDGKSINAGGHKVGL |
| 44 | 83.32 | 484.6031 (+3) | 484.6042 | 274-288 | VDGKSINAGGHKVGL |
| 45 | 83.13 | 526.7957 (+2) | 526.7965 | 278-288 | SINAGGHKVGL |
| 46 | 47.91 | 645.3458 (+1) | 645.3459 | 289-294 | ALELEA |

^*^A: N-terminal acetylated; ***C***: cysteine carboxyamidomethylated; **Q**: pyroglutamic acid form.

**Table S8. Sulfur-modified peptides found in hVDAC2 chymotryptic digest after DTT reduction and carboxyamidomethylation.**

Retention time, experimentally measured and calculated monoisotopic m/z of the molecular ions,position in the sequence and peptide sequence of modified fragments present in the tryptic digest of reduced and carboxyamidomethylated hVDAC2 are reported. All sequences were confirmed by MS/MS.

| **Frag. n.** | **Rt (min)** | **Monoisotopic m/z** | | **Position in the sequence** | **Peptide sequence** |
| --- | --- | --- | --- | --- | --- |
|  |  | **Measured** | **Calculated** |  |  |
| 1 | 82.51 | 808.8561 (+2) | 808.8565 | 130-142 | KRE***C***INLG**C**DVDF |
| 2 | 83.32 | 962.7771 (+3) | 962.7781 | 130-155 | KRE***C***INLG**C**DVDFDFAGPAIHGSAVF |
| 3 | 64.28 | 747.8359 (+2) | 747.8355 | 207-219 | **Q**KV**C**EDLDTSVNL |

**M**: methionine sulfoxide; **C**: cysteine oxidized to sulfonic acid; **Q**: pyroglutamic acid form.

**Table S9. Tryptic peptides found in hVDAC3 after DTT reduction and carboxyamidomethylation.**

Respective retention time, experimentally measured and calculated monoisotopic m/z of the molecular ions, position in the sequence and peptide sequence of fragments present in the tryptic digest of reduced and carboxyamidomethylated hVDAC3 are reported. All sequences were confirmed by MS/MS. These sequences were used to build the sequence coverage reported in Figure 5.

| **Frag.**  **n.** | **Rt**  **(min)** | **Monoisotopic m/z** | | **Position in the sequence** | **Peptide sequence** |
| --- | --- | --- | --- | --- | --- |
|  |  | **Measured** | **Calculated** |  |  |
| 1 | 42.66 | 685.7897 (+2) | 685.7894 | 2-12 | ****C***NTPTY***C***DLGK |
| 2 | 67.96 | 577.9682 (+3) | 577.9677 | 13-28 | AAKDVFNKGYGFGMVK |
| 3 | 56.97 | 622.3202 (+1) | 622.3195 | 16-20 | DVFNK |
| 4 | 70.33 | 487.9118 (+3) | 487.9114 | 16-28 | DVFNKGYGFGMVK |
| 5 | 65.19 | 429.7118 (+2) | 429.7126 | 21-28 | GYGFGMVK |
| 6 | 76.51 | 740.6682 (+3) | 740.6673 | 33-53 | TKS***C***SGVEFSTSGHAYTDTGK |
| 7 | 65.94 | 995.9267 (+2) | 995.9260 | 35-53 | S***C***SGVEFSTSGHAYTDTGK |
| 8 | 55.12 | 410.2136 (+2) | 410.2140 | 54-61 | ASGNLETK |
| 9 | 69.57 | 811.4081 (+2) | 811.4058 | 62-74 | YKV***C***NYGLTFTQK |
| 10 | 67.11 | 954.4420 (+2) | 954.4421 | 75-90 | WNTDNTLGTEISWENK |
| 11 | 72.64 | 720.0880 (+3) | 720.0876 | 91-110 | LAEGLKLTLDTIFVPNTGKK |
| 12 | 88.90 | 1508.0270 (+3) | 1508.0276 | 121-161 | D***C***FSVGSNVDIDFSGPTIYGWAVLAFEGWLAGYQMSFDTAK |
| 13 | 69.46 | 490.5965 (+3) | 490.5964 | 162-174 | SKLSQNNFALGYK |
| 14 | 62.54 | 627.8281 (+2) | 627.8275 | 164-174 | LSQNNFALGYK |
| 15 | 72.74 | 845.7346 (+3) | 845.7348 | 175-197 | AADFQLHTHVNDGTEFGGSIYQK |
| 16 | 72.25 | 733.3964 (+3) | 733.3960 | 198-218 | VNEKIETSINLAWTAGSNNTR |
| 17 | 58.53 | 429.1861 (+2) | 429.1859 | 225-230 | YMLD***C***R |
| 18 | 71.98 | 701.0652 (+3) | 701.0637 | 237-256 | VNNASLIGLGYTQTLRPGVK |
| 19 | 68.52 | 610.3358 (+3) | 610.3354 | 257-274 | LTLSALIDGKNFSAGGHK |
| 20 | 66.67 | 409.2012 (+2) | 409.2012 | 267-274 | NFSAGGHK |
| 21 | 63.61 | 934.4893 (+1) | 934.4880 | 275-283 | VGLGFELEA |

^*^***C***: N-terminal acetylated; ***C***: cysteine carboxyamidomethylated.

**Table S10. Sulfur-modified peptides found in hVDAC3 tryptic digest after DTT reduction and carboxyamidomethylation.**

Retention time, experimentally measured and calculated monoisotopic m/z of the molecular ions, position in the sequence and peptide sequence of modified fragments present in the tryptic digest of reduced and carboxyamidomethylated hVDAC3 are reported.

| **Frag. n.** | **Rt (min)** | **Monoisotopic m/z** | | **Position in the sequence** | **Peptide sequence** |
| --- | --- | --- | --- | --- | --- |
|  |  | **Measured** | **Calculated** |  |  |
| 1 | 60.77 | 437.7098 (+2) | 437.7103 | 21-28 | GYGFG**M**VK |
| 2 | 52.53 | 991.4069 (+2) | 991.4079 | 35-53 | S**C**SGVEFSTSGHAYTDTGK |
| 3 | 60.39 | 806.8889 (+2) | 806.8877 | 62-74 | YKV**C**NYGLTFTQK |
| 4 | 87.68 | 1513.3688 (+3) | 1513.3596 | 121-161 | D***C***FSVGSNVDIDFSGPTIYGWAVLAFEGWLAGYQ**M**SFDTAK |
| 5 | 54.24 | 437.1832(+2) | 437.1836 | 225-230 | Y**M**LD***C***R |

***C***: cysteine carboxyamidomethylated;**C**: cysteine oxidized to sulfonic acid; **M**: methionine sulfoxide.

**Table S11. Chymotryptic peptides found in hVDAC3 after DTT reduction and carboxyamidomethylation.**

Retention time, experimentally measured and calculated monoisotopic m/z of the molecular ions, position in the sequence and peptide sequence of fragments present in the chymotryptic digest of reduced and carboxyamidomethylated hVDAC3 are reported. These sequences were used to build the sequence coverage reported in Figure 5.

| **Frag.**  **n.** | **Rt**  **(min)** | **Monoisotopic m/z** | | **Position in the sequence** | **Peptide sequence** |
| --- | --- | --- | --- | --- | --- |
|  |  | **Measured** | **Calculated** |  |  |
| 1 | 46.14 | 797.3140 (+1) | 797.3134 | 2-7 | ****C***NTPTY |
| 2 | 82.39 | 1001.4583 (+2) | 1001.4559 | 2-18 | ****C***NTPTY***C***DLGKAAKDVF |
| 3 | 83.09 | 532.2928 (+2) | 532.2933 | 9-18 | DLGKAAKDVF |
| 4 | 84.76 | 571.7789 (+2) | 571.7792 | 32-41 | KTKS***C***SGVEF |
| 5 | 82.52 | 742.8654 (+2) | 742.8656 | 49-62 | TDTGKASGNLETKY |
| 6 | 63.49 | 675.8124 (+2) | 675.8128 | 76-87 | NTDNTLGTEISW |
| 7 | 54.92 | 692.3252 (+1) | 692.3255 | 82-87 | GTEISW |
| 8 | 91.59 | 873.4681 (+1) | 873.4682 | 88-95 | ENKLAEGL |
| 9 | 83.47 | 475.7809 (+2) | 475.7820 | 96-103 | KLTLDTIF |
| 10 | 70.83 | 709.3773 (+1) | 709.3772 | 98-103 | TLDTIF |
| 11 | 63.46 | 1019.5420 (+1) | 1019.5413 | 98-106 | TLDTIFVPN |
| 12 | 59.81 | 526.7488 (+2) | 526.7489 | 124-133 | SVGSNVDIDF |
| 13 | 65.20 | 835.8998 (+2) | 835.8996 | 124-139 | SVGSNVDIDFSGPTIY |
| 14 | 65.35 | 609.2670 (+1) | 609.2673 | 145-149 | AFEGW |
| 15 | 88.40 | 690.8130 (+2) | 690.8130 | 179-190 | **Q**LHTHVNDGTEF |
| 16 | 83.08 | 466.5529 (+3) | 466.5535 | 179-190 | QLHTHVNDGTEF |
| 17 | 82.30 | 510.2252 (+2) | 510.2256 | 182-190 | THVNDGTEF |
| 18 | 82.21 | 749.9092 (+2) | 749.9098 | 196-208 | **Q**KVNEKIETSINL |
| 19 | 83.08 | 758.4230 (+2) | 758.4230 | 196-208 | QKVNEKIETSINL |
| 20 | 92.01 | 878.4682 (+2) | 878.4679 | 196-210 | **Q**KVNEKIETSINLAW |
| 21 | 82.55 | 484.2334 (+2) | 484.2337 | 211-219 | TAGSNNTRF |
| 22 | 84.26 | 622.3562 (+1) | 622.3559 | 220-225 | GIAAKY^a^ |
| 23 | 76.39 | 452.2487 (+2) | 452.2489 | 234-242 | SAKVNNASL |
| 24 | 91.90 | 703.8864 (+2) | 703.8860 | 234-247 | SAKVNNASLIGLGY |
| 25 | 83.13 | 556.8425 (+2) | 556.8429 | 248-257 | TQTLRPGVKL |
| 26 | 92.28 | 589.8251 (+2) | 589.8249 | 258-268 | TLSALIDGKNF |
| 27 | 82.52 | 482.7581 (+2) | 482.7591 | 260-268 | SALIDGKNF |
| 28 | 83.18 | 413.2320 (+2) | 413.2331 | 269-277 | SAGGHKVGL |

*C: N-terminal acetylated; ***C***: cysteine carboxyamidomethylated. ^a^This peptideis common to all the isoforms and therefore its attribution to the hVDAC3 sequence it is not univocal.
